# New potentiometric sensors for methylphenidate detection based on host–guest interaction

**DOI:** 10.1186/s13065-019-0634-3

**Published:** 2019-10-14

**Authors:** Haitham AlRabiah, Mohammed Abounassif, Haya I. Aljohar, Gamal Abdel-Hafiz Mostafa

**Affiliations:** 1 Pharmaceutical Chemistry Department, College of Pharmacy, King Saud University, P.O. Box 2457, Riyadh, 11451 Saudi Arabia; 2 Micro-analytical Lab, Applied Organic Chemistry Department, National Research Center, Dokki, Cairo, Egypt

**Keywords:** Methylphenidate, β-CD, γ-CD, 4-*tert*-butylcalix[8]arene, Ionophore, Sensors, Potentiometry

## Abstract

The study aims to develop simple, sensitive, and selective methods for detecting methylphenidate in its bulk, dosage form and human urine. Sensing materials include β-cyclodextrin (β-CD), γ-cyclodextrin (γ-CD), and 4-*tert*butylcalix[8]arene as ionophores or electroactive materials have been used for construction of sensors 1, 2, and 3, respectively; Potassium tetrakis (4-chlorophenyl)borate (KTpClPB) as an ion additive was used and dioctyl phthalate as a plasticizer. The sensors displayed a fast, stable response over a wide concentration range of methylphenidate (8 × 10^−6^ M to 1 × 10^−3^ M) with 10^−6^ M detection limit over the pH range of 4–8. The developed sensors displayed a Near-Nernstian cationic response for methylphenidate at 59.5, 51.37, and 56.5 mV/decade for sensors β-CD, γ-CD, or 4-*tert*butylcalix[8]arene respectively. Validation of the proposed sensors is supported by high accuracy, precision, stability, fast response, and long lifetimes, as well as selectivity for methylphenidate in the presence of different species. Sensitive and practical sensors for the determination of methylphenidate in bulk, in pharmaceutical forms and urine were developed and validated for routine laboratory use. The results were comparable to those obtained by HPLC method.

## Introduction

**Fig. 1:**
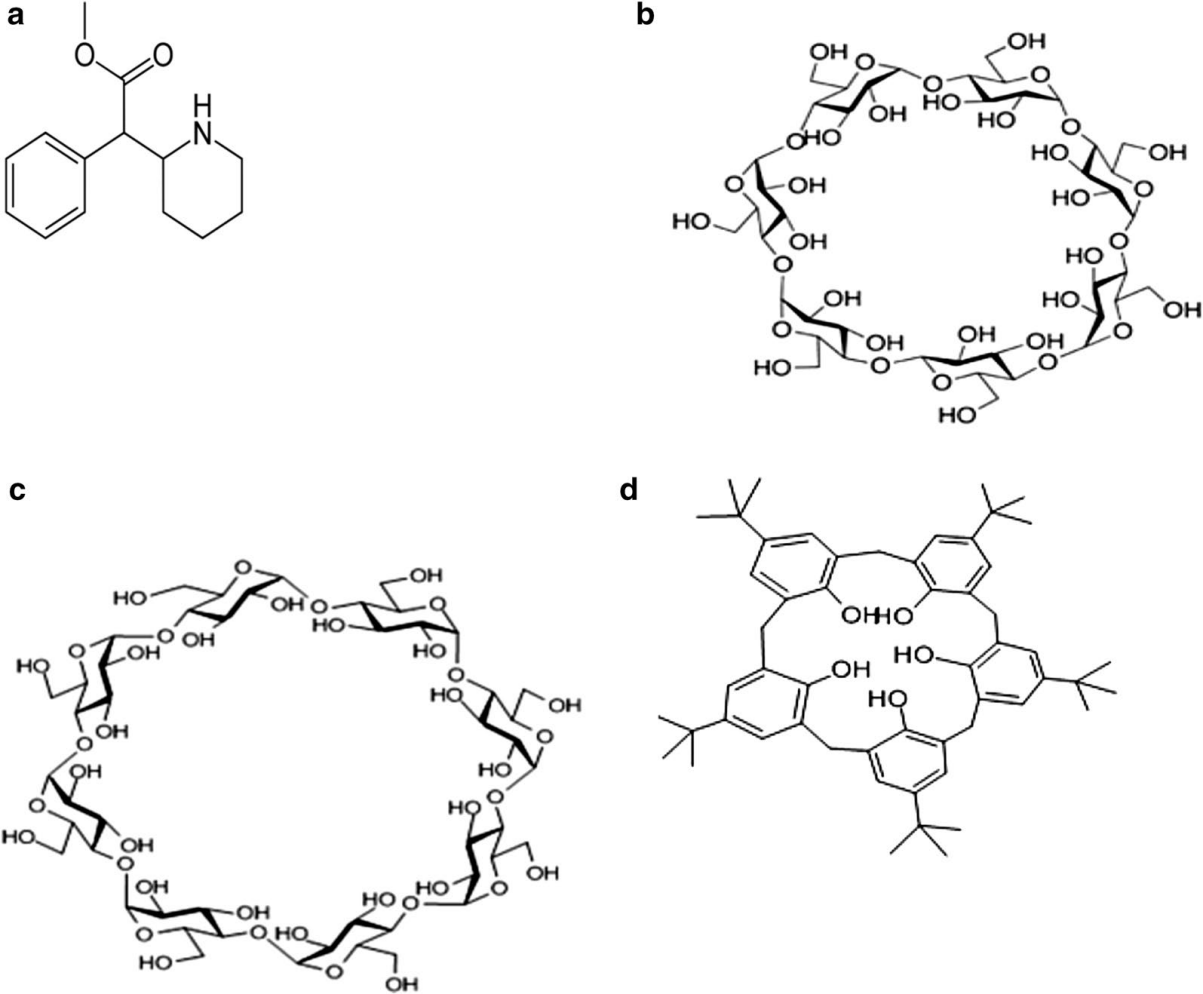
Chemical structure of **a** methylphenidate, **b** β -CD, **c** γ-CD, and **d** 4-*tert*-butylcalix[8]arene

Methylphenidate is a piperidine derivative that acts as an activator for the central nervous system used to treat hyperactivity and attention deficit. Hyperactivity is believed to be associated with reduced dopamine and norepinephrine functions in the brain; dopamine and norepinephrine are responsible for human executive functions, such as logic, inhibitory behavior, organization, problem solving and planning [1, 2]. The chemical nomenclature of methylphenidate is methyl 2-phenyl-2-(piperidin-2-yl) acetate and the structure is shown in Fig. 1a. Methylphenidate inhibits the reuptake of catecholamines by blocking dopamine and norepinephrine transport, which increases the concentration of catecholamines at their active sites [3].

Different analytical techniques for assaying of methylphenidate have been available, most of which rely on chromatographic methods [4–15] using HPLC-ultraviolet detection [4, 5], HPLC-fluorescence detection [6, 7], HPLC-chemiluminescence detection [8], HPLC-mass spectrometry [9–12], and enantiomeric resolution [13–15]. Most of these methods incorporate sample treatments steps and require expensive instruments. The lack of functional groups (–NH_2_, –OH, –COOH, –CO, –CHO, ….) attached to the main structure of the drug (responsible for chemical reactions by the compound) makes it chemical reactivity very limited. Therefore, detection of the drug using spectroscopic or electrochemical techniques are not widely used. Thus, we aimed to develop, for the first time, a cost effective potentiometric sensors for the detection of methylphenidate.

Polyvinyl chloride (PVC) membrane sensors are relatively inexpensive, simple, highly selective, with a fast response and represent one of the few techniques used for detection of both cation or anionic compounds [16, 17]. Moreover, the application of PVC membrane sensors in biological/medical matrices was previously described [18–20]. In addition, this technique is not affected by the presence of color or turbid samples [21].

The current study describes the applicability of either β-CD, γ-CD, or 4-*tert*-butylcalix[8]arene as ionophores and potassium tetrakis (4-chlorophenyl)borate as an ion additive to construct and develop new PVC membrane sensors for methylphenidate. The methods were then used for detecting methylphenidate in its bulk, dosage form and urine. The obtained results were compared with HPLC.

## Materials and methods

### Apparatus

All potentiometric measurements were performed at 25 ± 1 °C unless otherwise stated, using a HANNA pH 211 pH meter with methylphenidate indicator sensors in conjunction with a reference electrode (Merck) containing 10% (w/v) potassium nitrate in the outer compartment. The pH was measured using a combined Ross glass pH electrode.

The chromatographic assay of methylphenidate was carried out on Waters HPLC system (Milford, USA) equipped with a “Waters 1500 series HPLC pump, a Waters 2489 dual-wavelength UV detector, and a Waters 717 Plus autos ampler”. The chromatographic separation was achieved with an analytical C18 analytical column (125 mm × 4.6 mm internal diameter × 3 μm particle size) (Waters, Ireland) using a mixture of methanol: acetonitrile: acetate (pH 4.0) as mobile phase. The detection was carried out at 230 nm by UV detection [4].

### Reagents and materials

All chemicals were of analytical reagent grade and double distilled water were used throughout. High molecular weight PVC powder, dibutyl phthalate (DBP), dioctyl phthalate (DOP), *o*-nitrophenyloctyl ether (*o*-NPOE), and tetrahydrofuran (THF) of purity > 99% were obtained from Aldrich Chemical Company and methylphenidate HCl, β-CD, γ-CD, 4-*tert*-butylcalix[8]arene and KTpClPB were obtained from Sigma Chemical Company, Germany. Methylphenidate tablets (10 mg; Laboratories Rubio, S.A., 08755 Castellbisbal, Spain) and Ritalin, 10 mg MP, Novartis were obtained from a local pharmacy, Saudi Arabia. An appropriate amount of methylphenidate was dissolved in distilled water to prepare a 1 × 10^−2^ M solution. Working solutions of methylphenidate (1 × 10^−2^ –1 × 10^−6^ M) were prepared by serial dilution of the stock in distilled water. Acetate buffer solution of pH 5 was prepared using mixture of 0.05 M sodium acetate and acetic acid.

### Preparation of the MP-PVC membrane sensors

The ionophore materials (β-CD, γ-CD, or 4-*tert*-butylcalix [8]arene; 5 mg each) were combined with KTpClPB as an additive (5 mg) and thoroughly mixed with the PVC powder (190 mg), and 350 mg of the plasticizer (DBS, DOP, or *o*-NPOE) followed by addition of THF (5 mL) in glass Petri dishes (5 cm diameter). After mixing the constituents, the solvent was allowed to evaporate for about 20 h while the sensing membranes formed. The PVC master membranes were sectioned using a cork borer (10 mm diameter) and glued to a polyethylene tube (3 cm long, 8 mm i.d.) using THF [16, 17]. Glass electrode bodies were used and connected with a polyethylene tube at one end then the indicator electrode was filled with the internal standard solutions (the same volumes of 1 × 10^−2^ M aqueous solutions of methylphenidate and KCl). Ag/AgCl internal reference electrode (1.0 mm diameter) was used. The working electrode was conditioned by keeping it in a 1 × 10^−2^ M aqueous methylphenidate for 1 h and it was kept in diluted solution of methylphenidate after finishing the work.

### Effect of pH and response time

The pH of the investigated sensors at two concentrations of methylphenidate was assessed for the optimum pH relative to response to methylphenidate. The pH was controlled using a weak HCl or NaOH solution. The methylphenidate-PVC sensors were tested using two concentrations (0.001 M and 0.0001 M) of relative response to methylphenidate.

One of the most important factors that affect electrode characterization is the stability of potential reading of the developed sensors. The minimum time required to obtain the potential reading of a sensor after inserting the electrode into the methylphenidate test solution (increasing or decreasing the concentration) is the assessed as an average time.

### Procedure

The methylphenidate-PVC sensors were standardized by immersion in combination with a reference electrode in an electrochemical cell containing 9.0 mL acetate buffer of pH 5. Then, a 1.0 mL aliquot methylphenidate solution was added with constant stirring to obtain the final drug concentrations ranging from 10^−6^ to 10^−3^ M and the potential was recorded after each addition. Calibration graphs were then made by plotting the potentials as a function of −log[methylphenidate]. The extracted equation of each calibration line was used for the assay of solution with unknown methylphenidate concentration.

### Detection of methylphenidate in its dosage form

Ten tablets of methylphenidate (10 mg each) were weighed, crushed and blended in a mortar. An adequate amount (10 mg methylphenidate powder) was transferred into a 100 mL beaker, dissolved in distilled water, sonicated for approximately 10 min, filtered and collected in 100 mL measuring flask, and filled with water. Aliquots (5.0 mL) were moved into a 50 mL measuring flask, the pH was adjusted to 5 using acetate buffer, and the volume mad up with water. The potential of the formed solution was recorded using methylphenidate sensors in conjunction with a reference double junction electrode. The concentration was calculated from the previously constructed calibration equations using the different sensors. The potentials of the methylphenidate assay solution were recorded before and after the addition of a 1.0 mL of 1 × 10^−3^ M solution. The unknown concentration of methylphenidate was assessed using standard addition technique [16].

To prepare the reconstituted powder, a mixture was made with a fixed amount of methylphenidate powder (5 mg) and tablet ingredients starch, lactose, and magnesium stearate. The constituents were dissolved in water, sonicated for 15 min, filtered, and collected in a calibrated measuring flask. The unknown concentration was assessed to measure both recovery and accuracy.

### Determination of methylphenidate in urine

A urine sample was obtained from a healthy volunteer and spiked with 1 × 10^−5^ g/L methylphenidate. The prepared sample was centrifuged at 3000 ppm for 8 min. Then the clear upper layer was analysis as recommended procedure.

## Results and discussion

### Mechanism of sensing membrane

Ion-selective membrane sensors are based on membrane selectivity (recognitions of target ions) across the membrane interface between the sample and membrane phase, which generates a potential difference [22]. The mechanism of selectivity is dependent on various mechanism [23] based on a complexation reaction between the analyte (guest ion) and a carrier referred to as host, sensing material or ionophore: (1) the size of the carrier compound, should be suitable enough to accumulate the target ions (analyte or gust) and (2) the number of donor atoms in the guest or analyte, which helps the formation of a coordination reaction between the guest and host [24].

Cyclodextrins (CDs) are commonly used as receptors in host–guest inclusion complexes [25, 26]. Additionally, 4-*tert*-butylcalix[8]arenes are well known as selective ligands for many different ions [27]. 4-*tert*-butylcalix[8]arenes form stable inclusion complexes (host–guest interaction) through dipole–dipole interactions and therefore different ionic selective membrane can be made [28–30]. CDs have a large cyclic-like structure present as a cylindrical funnel with an upper, wide rim and a lower, narrow rim (Fig. 1b, c). The upper rim in the CDs is composed of secondary alcohols, while the lower rim consists of primary alcohols [25], which allow the coordination between the carrier and guest.

The degree of complexation between host and guest is based on the size of the carrier (ionophore). The host–guest interactions are based on different forces e.g. formation of hydrogen bonds, hydrophobic interactions and van der Waals force [31]. The carriers used in the present investigation are β-CD, γ-CD, and 4-*tert*-butylcalix[8]arene. β-CD and γ-CD are 7-membered and 8-membered sugar ring molecules, respectively. On the other hand, methylphenidate has donor atoms (oxygen and nitrogen) that assist the coordination reaction between host and guest. In addition, methylphenidate has a positive charge, which also assists the coordination reaction between guest and host, through the formation of a flexible inclusion complex reaction.

### The effect of the additive

The additive in membrane composition plays a significant role in the sensing mechanism; the additive is employed to produce ionic sites through the membrane material. This procedure improves the analytical behavior of the investigated membrane, which becomes more ionic (cationic or anionic) [16, 17]. In this case study, the addition of KTpClPB converts the neutral site of the carrier to a cationic site, which allows the detection of cations (methylphenidate ions) by reducing anionic interferences, thus increasing selectivity towards the target analyte [27]. It also enhances the ion-exchange response, which increases the sensitivity of the proposed sensors [24]. In this study, we used KTpClPB, which allows the carrier to produce cationic sites through the sensing membrane, and it acts as an anionic excluder in the other direction, reducing the selectivity. Therefore, the additive increases the sensitivity and increase selectively of the proposed PVC sensors towards the proposed drug [24, 27]. The addition of additive KTpClPB from 1 mg to 7 mg was studied, as the concentration of additive increase the sensitivity of the methylphenidate sensors increase till 5 mg, upon increasing of KTpClPB till 7 mg the sensitivity is remaining constant. Therefore, 5 mg was chosen as the optimum concentration of the additive (KTpClPB). The results are listed in Table 1.

**Table 1:** Optimization of the PVC membrane composition

No.	Plasticizer (350 mg)	KTPB (mg)	Sensor 1		Sensor 2		Sensor 3	
			Slope	Range	Slope	Range	Slope	Range
1	DBS	5	50	1 × 10^−3^ to 1 × 10^−5^	45	1 × 10^−3^ to 1 × 10^−5^	47	1 × 10^−3^ to 1 × 10^−5^
2	DOP	5	59.5	1 × 10^−3^ to 8 × 10^−6^	51.5	1 × 10^−3^ to 8 × 10^−6^	56.5	1 × 10^−3^ to 8 × 10^−6^
3	o-NOPE	5	59.0	1 × 10^−3^ to 8 × 10^−6^	51.0	1 × 10^−3^ to 8 × 10^−6^	56.5	1 × 10^−3^ to 8 × 10^−6^
4	DOP	0	37	1 × 10^−3^ to 2 × 10^−5^	35	1 × 10^−3^ to 2 × 10^−5^	40	1 × 10^−3^ to 2 × 10^−5^
5	DOP	1	50	1 × 10^−3^ to 2 × 10^−5^	47	1 × 10^−3^ to 2 × 10^−5^	48	1 × 10^−3^ to 2 × 10^−5^
6	DOP	3	53	1 × 10^−3^ to 1 × 10^−5^	49	1 × 10^−3^ to 1 × 10^−5^	52.5	1 × 10^−3^ to 1 × 10^−5^
7	DOP	5	59	1 × 10^−3^ to 8 × 10^−6^	51.5	1 × 10^−3^ to 8 × 10^−6^	56.5	1 × 10^−3^ to 8 × 10^−6^
8	DOP	7	59	1 × 10^−3^ to 8 × 10^−5^	51.5	1 × 10^−3^ to 8 × 10^−5^	56.5	1 × 10^−3^ to 8 × 10^−5^

Sensor 1 (β-CD), Sensor 2 (γ-CD) and Sensor 3 (calaxirene)

### The effect of plasticizers

Methylphenidate-PVC membrane sensors were assessed for the effect of using different plasticizers in relation to their analytical characteristics. The three plasticizers were DBP, DOP, and *o*-NPOE. The role of the plasticizers in the manufacturing of such PVC membranes is to produce a plastic membrane that is flexible and homogeneous to assist ion exchange through the membrane. DOP and o-NPOE were observed to be suitable plasticizers, accessible and available mediators for methylphenidate sensors compared with DBP. The solvation of the ionophores by DOP and o-NPOE seemed to be suitable for the construction of the sensors; however, in the case of *o*-NPOE the nature of the membrane is oily and therefore it is not easily handle. Therefore, the best results were acquired using DOP (ε = 7) compared with *o*-NPOE (ε = 24). In addition, different quantities of plasticizer (250, 300, 350 and 400 μL) were tested. The rigidity of the membranes made with 250, 300 μL plasticizer was very low, and therefore the handling of the membrane is harder, whereas with 350 or 400 μL, this was better handled. Thus 350 μL was used as the most appropriate quantity of plasticizer. The effect of different plasticizer on the membrane composition was listed in Table 1. The results indicate that DOP was better compared with o-NPOE and DBS.

### Influence of pH and response time

**Fig. 2:**
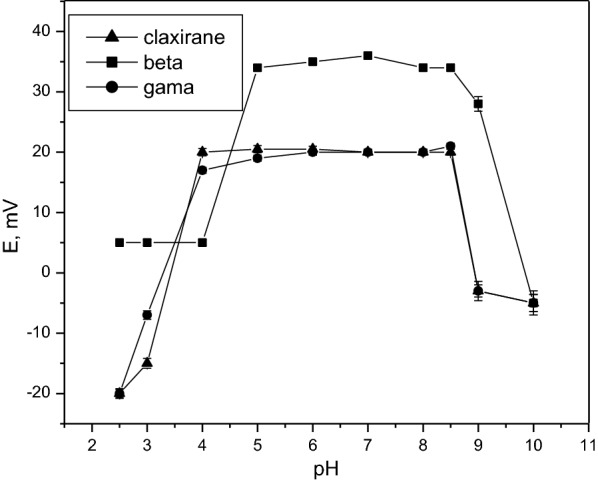
Effect of pH over the proposed sensors

The pH diagram for the investigated sensors had constant slopes (51.37, 59.4, and 56.3 mV/decade for sensors 1, 2, and 3, respectively) over the pH range 4 -8, as presented in Fig. 2. At higher pH (pH > 8), the potential decreases because the amount of un-protonated methylphenidate increases at higher pH (pKa = 8.9) [32]. Figure 2 shows that the potential was constant in the pH range of 4–8. Different buffer solutions were tested (phosphate, acetate) over the optimum pH range (4–8). Acetate buffer (pH 5) appeared to be the best performing buffer; therefore, acetate buffer was used for all experiments.

**Fig. 3:**
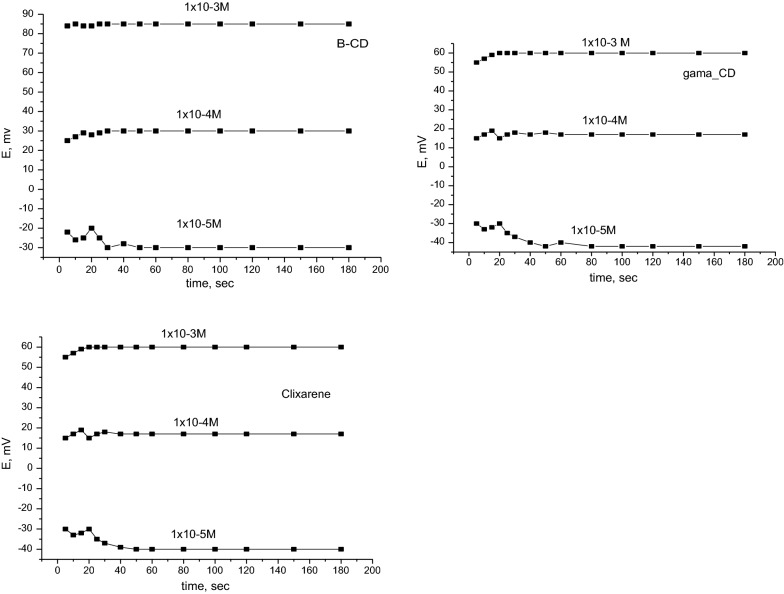
Response time of the methylphenidate sensors for the changes in the concentration (1 × 10^−3^, 1 × 0^−4^, and 1 × 10^−5^ M)

As presented in Fig. 3 the sensor response time [33] was 25 s whereas the potential reading of the proposed sensors before 20 s was unstable after 25 s the electrode potential was stable, therefore the response time was 25 s. The repeatability of the response was approximately within ± 1 mV for each test concentration. The lifetime of the developed sensors were approximately 8 weeks (i.e., the period over which response was stable) where the RSD of the sensors was less than 3%. During 2 months, the membrane showed reproducible results, indicating that the PVC sensors were stable for the indicated lifetime. After 2 months, the new section of the membrane showed reproducible of less than 4%.

### Interference studies

The impact of various ions on the selectivity of the developed sensors was investigated. The $K_{A, B}^{Pot}$ of the proposed sensors was studied according the IUPAC recommendations using either separate or mixed solution method [33, 34] at pH 5. $K_{A, B}^{Pot}$ was estimated for the separate solution method according to Eq. (1):

1 $$\log K_{A, B}^{pot} = \frac{{E_{B - } E_{A} }}{S} + \left[ {1 - \frac{{Z_{A} }}{{Z_{B} }} } \right]\log a_{A}$$

where E_A_ and E_B_ are the potential readings of methylphenidate and interfering ion concentration (1 × 10^−3^ M each), respectively; $a_{A}$ and $a_{B}$ are the activities of methylphenidate and interfering species, respectively; Z_A_ and Z_B_ are the charge of methylphenidate and interfering species, respectively; and *S* is the slope of the graph (mV/decade). The selectivity coefficient values for the mixed solution method were estimated according to Eq. (2):

2 $$K_{A, B}^{pot} = \frac{{\left( {a^{\prime}_{A} - a_{A} } \right)}}{{a_{B} }}$$

where $$a^{\prime}_{A}$$ is the known activity of a primary ion that is added to a known solution that has a fixed activity ($$a_{A} )$$ of primary ions, and the corresponding potential change (ΔE) is recorded. Another test, a solution of an interfering ion $$\left( {a_{B} } \right)$$ is added to the known solution until the same potential change (ΔE) was recorded. Table 2 shows the results of interference tests. The results show reasonable selectivity for methylphenidate in the presence of most investigated interfering species. These data show that $$K_{A, B}^{Pot}$$ had low values, indicating high selectivity of the proposed sensors to methylphenidate.

**Table 2:** Potentiometric selectivity coefficients of some interfering ions, using methylpheinadte-PVC sensors

Interferent, J	$\mathop K\nolimits_{MP,B}^{Pot}$ Sensor 1	$\mathop K\nolimits_{MP,B}^{Pot}$ Sensor 2	$\mathop K\nolimits_{MP,B}^{Pot}$ Sensors 3
Na^+^	1 × 10^−3^	2 × 10^−3^	1.8 × 10^−3^
K^+^	2 × 10^−2^	2 × 10^−3^	1.7 × 10^−2^
Ca^2+^	1.9 × 10^−3^	1.7 × 10^−3^	2.0 × 10^−3^
Fe^+^	2.0 × 10^−3^	1.8 × 10^−3^	1.9 × 10^−3^
Acetate	1.8 × 10^−3^	1.8 × 10^−3^	1.9 × 10^−3^
Phosphate	2 × 10^−3^	1.7 × 10^−3^	1.9 × 10^−3^
Citrate	2 × 10^−3^	1.8 × 10^−3^	2.0 × 10^−3^
benzoate	2 × 10^−3^	1.8 × 10^−3^	2.0 × 10^−3^
Caffeine	3.7 × 10^−3^	4.0 × 10^−3^	3.3 × 10^−3^
Glycine	2.8 × 10^−2^	2.7 × 10^−2^	2.8 × 10^−2^
l-Cysteine	2.7 × 10^−2^	2.8 × 10^−2^	2.7 × 10^−2^
Tryptophan	2 × 10^−3^	2.1 × 10^−3^	2.1 × 10^−3^
Starch	3.8 × 10^−3^	4.8 × 10^−3^	4.5 × 10^−3^
Magnesium stearate	3.8 × 10^−3^	4.0 × 10^−3^	3.5 × 10^−3^
Lactose monohydrate	3.9 × 10^−3^	4.7 × 10^−3^	3.5 × 10^−3^
Glucose	3.7 × 10^−2^	4.3 × 10^−2^	3.3 × 10^−2^
Microcrystalline cellulose	3.5 × 10^−3^	4.7 × 10^−3^	4.6 × 10^−3^

Sensor 1 (β-CD), Sensor 2 (γ-CD) and Sensor 3 (calaxirene)

### Characteristics of the developed sensors

The potentiometric features of the developed sensors for methylphenidate utilizing: β-CD, γ-CD, and 4-*tert*-butylcalix[8]arene ionophores as sensing carriers were evaluated according the IUPAC guidelines. Table 2 shows the results. The least squares equations of the calibration graphs are constructed in the general form:

3 $$E\left( {mV} \right) = Slog\left[ {MP} \right] + intercept$$

where *E* is the electrode potential and *S* is the slope of the calibration line (59.4 ± 1, 51.37 ± 1, and 56.5 ± 1 mV for sensors 1, 2, and 3, respectively); the intercept values were 220.45 ± 1, 216.58 ± 1, and 248.17 ± 1 for the three sensors, respectively.

### Validation of the method

#### Limits of detection and quantification

**Fig. 4:**
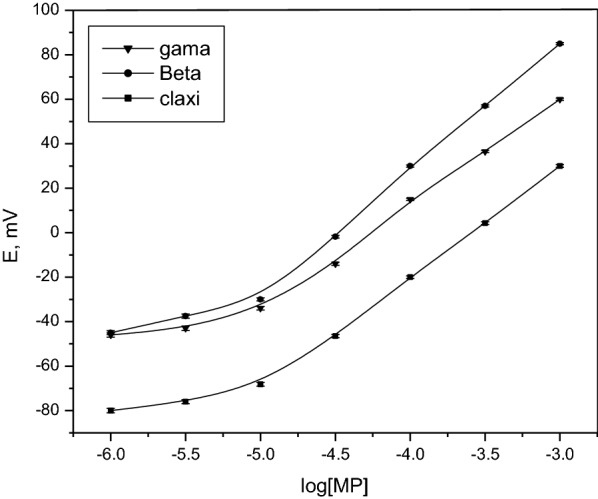
Calibration curve of the proposed sensors

The calibration plots of methylphenidate sensors were constructed by measuring the potential against the negative logarithmic of methylphenidate concentration. Each point in the calibration plot was the average of five measurements [35]. The measured potential was plotted against the -log concentration to establish the calibration line; r^2^ (correlation coefficient) was determined for the plot. The calibration range was 1 × 10^−3^ to 10^−6^ M for sensors 1, 2, and 3 over the optimal pH range (pH 4–8). The lower limit of detection (LOD) and quantification (LOQ) were calculated according the IUPAC guidelines [33]. The LOD values were 7 × 10^−6^, 7.5 × 10^−6^, and 7 × 10^−6^ M for the three sensors (1, 2, and 3, respectively), whereas the LOQ was 8 × 10^−6^ M for all sensors (Fig. 4).

#### Accuracy

The accuracy of the investigated sensors was expressed as the recovery (%) and was computed by calculating the measured concentration relative to the actual concentration in an acetate buffer (pH 5). The recovery was calculated according to Eq. (4):

4 $$Recovery\,\left( \% \right) = \left( { \frac{Measured\, concentration}{Added\, concentration}} \right) \times 100\%$$

The average recoveries (accuracies) within the same day (intra-day) of 26.09 μg/mL methylphenidate were 100.74%, 100.26%, and 101.48% for sensors 1, 2, and 3, respectively (Table 3). The average recoveries over different through diverse days (inter-day) were 97.43%, 97.1%, and 100.23% for sensors 1, 2, and 3, respectively (Table 4).

**Table 3:** Analytical parameters of methylphenidate-PVC sensors

Parameter	Sensor 1	Sensor 2	Sensor 3
Slope, (mV/decade)	59.4 ± 0.5	51.37 ± 0.5	56.5 ± 0.5
Intercept, mV	220.45 ± 0.5	216.58 ± 0.5	248.17 ± 0.5
Calibration range	8 × 10^−6^–1 × 10^−3^	8.0 × 10^−6^–1 × 10^−3^	8.0 × 10^−6^–1 × 10^−3^
STE YX	0.816	2.85	0.816
SE slope	0.5574	2.02	0.5574
SE Intercept	2.357	8.25	2.357
Correlation coefficient, (r^2^)	0.999	0.999	0.996
Lower limit of quantification (LOQ), M	8 × 10^−6^	8 × 10^−6^	8 × 10^−6^
Lower limit of detection (LOD), M	7.5 × 10^−6^	7 × 10^−6^	7 × 10^−6^
Response time for 1 × 10^−3^ M solution, (S)	25 ± 0.5	25 ± 0.5	25 ± 0.5
Working pH range	4–8	4–8	4–8

Sensor 1 (β-CD), Sensor 2 (γ-CD) and Sensor 3 (calaxirene)

*SE* standard error

**Table 4:** Day to day reproducibility of methylphenidate using methylphenidate -PVC membrane sensors

Parameter	Methylphenidate (26.9 μg/mL) Within-day			Methylphenidate (26.9 μg/mL) Within-days		
	Sensor 1	Sensor 2	Sensor 3	Sensor 1	Sensor 2	Sensor 3
Found, μg/mL	27.10	26.97	27.3	26.21	26.12	26.96
R, %	100.74	100.26	101.48	97.43	97.1	100.23
SD	0.65	0.59	0.63	0.65	0.59	0.63
RSD	2.39	2.19	2.31	2.47	2.26	2.34

n = 5

Sensor 1 (β-CD), Sensor 2 (γ-CD) and Sensor 3 (calaxirene)

#### Precision

The precision of the developed methods was tested [35] by performing the analysis on the same day and over different days for 26. 9 μg/mL methylphenidate (repeated five times within one day and within three days, respectively). The five repeated concentrations were used to calculate intra-day (through day) and inter-day precision. The intra-day precision values (expressed as % RSD) were 2.39%, 2.19%, and 2.33% for sensors 1, 2, and 3, respectively, whereas inter-day precision was assessed as 2.47%, 2.26%, and 2.34% for sensors 1, 2, and 3, respectively. All precision values were within the acceptable range, and the results are summarized in Table 4; all results are in the acceptable range.

#### Ruggedness and robustness

The ruggedness of the method [35] was assessed by measuring different concentrations by two different analysts and instruments on different days. The % RSD values were < 3%, representing that the developed methods are very rugged. The measured data also demonstrate that the suggested procedure is highly accurate. Changes of up to 10% from the optimum measuring conditions did not affect the response. The optimum pH value was 5 and the methods were highly robust in the optimum pH range (4–8). As presented in Table 5, the suggested procedure is highly ruggedness. On the other hand, the robustness of the investigated sensors was assessed during the day and different days at the optimum condition of the investigated sensors. The recovery during the day was 100.74%, 100.26%, 101.33% whereas RSD was 2.38%, 2.18%, and 2.21% for sensor 1, 2, and 3, respectively. Whereas the recovery during different days was 98.33%, 97.1%, 99.23%, while RSD was 2.5%, 2.4% and 2.7%, respectively. Results of ruggedness and robustness of the methylphenidate sensors are presented in Table 5.

**Table 5:** Ruggedness and Robustness of the methylphenidate sensors

Parameters	Sensor 1		Sensor 2		Sensor 3	
	Recovery, %	RSD, %	Recovery, %	RSD, %	Recovery, %	RSD, %
Operators 1	98.52	2.2	98.0	2.16	99.24	2.45
2	98.33	2.3	98.5	3.34	99.21	2.75
Instrument 1^a^	98.51	2.3	98.0	2.53	99.12	2.62
2	98.41	2.4	97.5	3.64	98.51	2.53
Change in day						
Intra-day	100.74	2.38	100.26	2.18	101.33	2.21
Inter-day	98.33	2.5	97.1	2.4	99.23	2.7
Life time during						
After 8 weeks	97.5	3.9	97.0	3.8	98.0	3.96

^a^Comparison between two instrument (HANNA pH 211 and WTW pH/mV meter (model 523; 8120 Weilheim, Germany

### Application of methylphenidate-PVC sensors

The application of methylphenidate-PVC sensors for the quantification of methylphenidate in its pharmaceutical form was investigated by examining the recovery of a known concentration of methylphenidate in standard solutions. The assay of 2.69 to 2697.7 μg/mL methylphenidate solutions (five replicates for each) was examined using the sensors. The recovery data showed that these methods are accurate (Table 6). The applicability of the methylphenidate sensors for quantifying methylphenidate was further examined by studying the determination of an exact concentration of methylphenidate in a synthetic laboratory powder tablet containing all tablet constituents. The accuracy using the sensors were 98.6%, 98.4% and 99.2% (with %RSD values of 1.80%, 2.23%, and 2.22%), respectively. The results confirmed that the proposed methods are highly accurate and precise. The final step was to assess the methylphenidate in its dosage form using the three sensors. The results are presented in Table 6. The results confirmed the precision and accuracy of the investigated methods. The results for the determination of methylphenidate in its dosage form were compared with the analysis results using published HPLC methodology [4] (Table 7). The data suggest that the sensors provide a high degree of accuracy and precision matching the performance of the HPLC method [4]. The accuracy of the three proposed methods and the reported HPLC method were compared using |t|2 for P = 0.05 and n = 5, resulting in |t|2 between 0.14 and 1.05. These values were lower than the tabulated value (|t|2 = 3.36) [35], indicating that the suggested sensors are as accurate as the reported HPLC method. The precision of the sensors and the reported HPLC method were compared using two-tailed F test. The values for a significant difference were in the range of 1.29–1.77, which is lower than the tabulated F value (6.38) [34]. These results indicate that the two methods are equally accurate. The proposed sensors was used for the assay of methylphenidate in urine samples with good accuracy and precision. The results are presented in Table 8.

**Table 6:** Determination of methylphenidate using the proposed PVC membrane sensors

Added conc., μg/mL	Sensors 1			
	2.69	26.9	269.7	2697.7
Measured	2.64	26.76	267.69	2695.1
R, %	98.14	99.47	99.25	99.90
SD	0.09	0.64	4.84	56.86
RSD, %	3.41	2.39	1.81	2.11
Sensor 2				
Added Conc., μg/mL	2.69	26.9	269.7	2697.7
Measured	2.63	26.36	264.95	2663.9
R, %	97.76	97.99	98.23	98.72
SD	0.1	0.66	5.91	52.72
RSD, %	3.80	2.51	2.23	1.96
Sensor 3				
Added conc., μg/mL	2.69	26.9	269.7	2697.7
Measured	2.64	26.36	265.17	2649.9
R, %	98.14	97.99	98.51	98.21
SD	0.1	0.64	5.89	50.1
RSD, %	3.78	2.43	2.22	1.89

R %: recovery %, SD; standard deviation, RSD %: relative standard deviation %

Sensor 1 (β-CD), Sensor 2 (γ-CD) and Sensor 3 (calaxirene)

**Table 7:** Determination of methylphenidate in some pharmaceutical preparations using the membrane sensors

Preparation	Parameter	Sensor 1	Sensor 2	Sensor 3	HPLC
Synthetic form, 5 mg	Measured	4.93	4.92	4.96	4.97
	R, %	98.6	98.4	99.20	99.4
	SD	0.09	0.11	0.11	0.13
	RSD, %	1.83	2.23	2.22	2.61
Ritalin tablet (10 mg)					
	Measured	9.92	9.92	9.8	9.9
	R, %	98.0	98.5	98	99
	SD	0.19	0.19	0.22	0.25
	RSD	1.9	1.95	2.26	2.55
T_test_		0.14	0.14	0.71	
F_test_		1.73	1.73	1.29	
Methylphenidate tablet (10 mg)					
	Measured	9.91	9.85	9.81	9.85
	R, %	99.1	98.5	98.1	98.5
	SD	0.18	0.19	0.22	0.24
	RSD, %	1.81	1.93	2.24	2.44
T_test_		0.49	0.5	1.05	
F_test_		1.19	1.5	1.77	

R %: recovery %, SD; standard deviation, RSD %: relative standard deviation %

**Table 8:** Determination of methylphenidate in spiking urine sample using the proposed sensors

Urine sample	Sensor 1	Sensor 2	Sensor 3	HPLC
Recovery, %	98.5	98.0	97.5	99.0
SD	0.084	0.092	0.081	0.077
RSD, %	3.2	3.5	3.1	2.9

## Conclusions

Three novel PVC membrane sensors for methylphenidate were constructed, optimized and validated. The investigated sensors used β-CD, γ-CD or 4-tert-butylcalix[8]arene as ionophores (electroactive materials), in the presence of DOP as a plasticizer and KTpClPB as an additive dispersed in a PVC matrix. The sensors demonstrate a fast, accurate, selective, and Near-Nernstian response over a wide methylphenidate concentration range in the pH range between 4 and 8. The detection of methylphenidate using the developed methods showed high accuracy and precision. β-CD show the best near-Nernstian behavior (59.5 mV) compared with γ-CD (51.37 ± 0.5) and claxiraine (56.5 ± 0.5). The determination of methylphenidate using the developed sensors was comparable with reported HPLC methodology. The developed sensors were successfully used to detect methylphenidate in bulk, its formulation, and urine and therefore the method can be used in routine quality-control laboratories and urine sample.

## Supplementary information

**Additional file 1.** Raw data of calibration curve, pH and response time.

## Data Availability

All data and material analyzed or generated during this investigation are included in this published article

## Abbreviations

β-CD: β-cyclodextrin
γ-CD: γ-cyclodextrin
KTpClPB: potassium tetrakis (4-chlorophenyl)borate
PVC: polyvinyl chloride
DBP: dibutyl phthalate
DOP: dioctyl phthalate
*o*-NPOE: *o*-nitrophenyloctyl ether
THF: tetrahydrofuran
CDs: cyclodextrins
