# Supplementary material for: New potentiometric sensors for methylphenidate detection based on host–guest interaction
Source: BMC Chem. 2019 Oct 14;13(1):121. doi: 10.1186/s13065-019-0634-3 (PMC6792245; doi:10.1186/s13065-019-0634-3)
Supplement: Supplementary file 1 — Additional file 1. Raw data of calibration curve, pH and response time. [file 13065_2019_634_MOESM1_ESM.docx]

**Additional file**

**Calibration data**

| **Conc, M** | **Calxi** | **SD** | **β-CD** | **SD** | **γ-CD** | **SD** |
| --- | --- | --- | --- | --- | --- | --- |
| 1x10-6 | -6 | 1 | -45 | 0.9 | -46 | 1 |
| 5x10-6 | -5.5 | 0.9 | -37.5 | 0.9 | -43 | 1 |
| 1x10-5 | -5 | -0.8 | -30 | 0.7 | -34 | 0.8 |
| 5x10-5 | -4.5 | 0.7 | -1.8 | 0.6 | -14 | 0.7 |
| 1x10-4 | -4 | 0.6 | 30 | 0.5 | 15 | 0.5 |
| 5x10-4 | -3.5 | 0.6 | 57 | 0.5 | 36.5 | 0.5 |
| 1x10-3 | -3 | 0.6 | 85 | 0.5 | 60 | 0.5 |

**Effect of PH**

| **pH** | **Calxi** | **SD** | **β-CD** | **SD** | **γ-CD** | **SD** |
| --- | --- | --- | --- | --- | --- | --- |
| 2.5 | -20 | 0.7 | 5 | 0.5 | -20 | 0.8 |
| 3 | -15 | 0.8 | 5 | 0.5 | -7 | 0.7 |
| 4 | 20 | 0.6 | 5 | 0.5 | 17 | 0.5 |
| 5 | 20.5 | 0.6 | 34 | 0.5 | 19 | 0.5 |
| 6 | 20.5 | 0.5 | 35 | 0.5 | 20 | 0.5 |
| 7 | 20 | 0.5 | 36 | 0.6 | 20 | 0.5 |
| 8 | 20 | 0.5 | 34 | 0.6 | 20 | 0.5 |
| 8.5 | 20 | 0.5 | 34 | 0.6 | 21 | 0.5 |
| 9 | -3 | 1.6 | 28 | 1.2 | -3 | 1 |
| 10 | -5 | 1.4 | -5 | 2 | -5 | 1.4 |

**Response time (Calxi)**

| **Response time, sec** | **1x10^-5^M** | **1x10^-4^M** | **1x10^-3^M** |
| --- | --- | --- | --- |
| 5 | -30 | 15 | 55 |
| 10 | -33 | 17 | 57 |
| 15 | -32 | 19 | 59 |
| 20 | -30 | 15 | 60 |
| 25 | -35 | 17 | 60 |
| 30 | -37 | 18 | 60 |
| 40 | -39 | 17 | 60 |
| 50 | -40 | 17 | 60 |
| 60 | -40 | 17 | 60 |
| 80 | -40 | 17 | 60 |
| 100 | -40 | 17 | 60 |
| 120 | -40 | 17 | 60 |
| 150 | -40 | 17 | 60 |
| 180 | -40 | 17 | 60 |

**Response time : β-CD**

| **Response time, sec** | **1x10^-5^M** | **1x10^-4^M** | **1x10^-3^M** |
| --- | --- | --- | --- |
| 5 | -22 | 25 | 84 |
| 10 | -26 | 27 | 85 |
| 15 | -25 | 29 | 84 |
| 20 | -20 | 28 | 84 |
| 25 | -25 | 29 | 85 |
| 30 | -30 | 30 | 85 |
| 40 | -28 | 30 | 85 |
| 50 | -30 | 30 | 85 |
| 60 | -30 | 30 | 85 |
| 80 | -30 | 30 | 85 |
| 100 | -30 | 30 | 85 |
| 120 | -30 | 30 | 85 |
| 150 | -30 | 30 | 85 |
| 180 | -30 | 30 | 85 |

**Response time: γ-CD**

| **Response time, sec** | **1x10^-5^M** | **1x10^-4^M** | **1x10^-3^M** |
| --- | --- | --- | --- |
| 5 | -30 | 15 | 55 |
| 10 | -33 | 17 | 57 |
| 15 | -32 | 19 | 59 |
| 20 | -30 | 15 | 60 |
| 25 | -35 | 17 | 60 |
| 30 | -37 | 18 | 60 |
| 40 | -40 | 17 | 60 |
| 50 | -42 | 18 | 60 |
| 60 | -42 | 17 | 60 |
| 80 | -42 | 17 | 60 |
| 100 | -42 | 17 | 60 |
| 120 | -42 | 17 | 60 |
| 150 | -42 | 17 | 60 |
| 180 | -42 | 17 | 60 |
